# Tixagevimab/Cilgavimab as pre-exposure prophylaxis against SARS-CoV-2 in patients with hematological malignancies

**DOI:** 10.3389/fonc.2023.1212752

**Published:** 2023-06-22

**Authors:** Francesco Angotzi, Marco Petrella, Tamara Berno, Gianni Binotto, Giorgia Bonetto, Antonio Branca, Marco Carraro, Chiara Adele Cavaretta, Alessandro Cellini, Fabio D’Amore, Laura Forlani, Ilaria Gianesello, Carmela Gurrieri, Silvia Imbergamo, Federica Lessi, Antonio Maroccia, Federica Mazzetto, Laura Pavan, Sara Pezone, Francesco Piazza, Stefano Pravato, Valeria Ruocco, Greta Scapinello, Fabrizio Vianello, Renato Zambello, Ivan Zatta, Simone Zoletto, Andrea Padoan, Livio Trentin, Andrea Visentin

**Affiliations:** ^1^Department of Medicine, Hematology and Clinical Immunology Unit, University of Padova, Padova, Italy; ^2^Department of Integrated Diagnostic Medicine, Laboratory Medicine Unit, University of Padova, Padova, Italy

**Keywords:** SARS-CoV-2, COVID-19, Tixagevimab/Cilgavimab, hematological malignances, monoclonal antibodies (mAbs)

## Abstract

The approved combination of Tixagevimab/Cilgavimab has been shown to decrease the rate of symptomatic SARS-CoV-2 infection in patients at increased risk of inadequate response to vaccination. However, Tixagevimab/Cilgavimab was tested in a few studies that included patients with hematological malignancies, even if this population has shown an increased risk of unfavorable outcomes following infection (with high rates of hospitalization, intensive care unit admission, and mortality) and poor significant immunization following vaccines. We performed a real-life prospective cohort study to evaluate the rate of SARS-CoV-2 infection following pre-exposure prophylaxis with Tixagevimab/Cilgavimab in anti-spike seronegative patients compared to a cohort of seropositive patients who were observed or received a fourth vaccine dose. We recruited 103 patients with a mean age of 67 years: 35 (34%) received Tixagevimab/Cilgavimab and were followed from March 17, 2022, until November 15, 2022. After a median follow-up of 4.24 months, the 3-month cumulative incidence of infection was 20% versus 12% in the Tixagevimab/Cilgavimab and observation/vaccine groups respectively (HR 1.57; 95% CI: 0.65-3.56; p = 0.34). In this study, we report our experience with Tixagevimab/Cilgavimab and a tailored approach to SARS-CoV-2 infection prevention in patients with hematological malignancies during the SARS-CoV-2 omicron surge.

## 1 Introduction

Patients with hematological malignancies who develop coronavirus infectious dis-ease (COVID-19) following infection by severe acute respiratory syndrome coronavirus 2 (SARS-CoV-2) usually display an increased risk of hospitalization and intensive care unit (ICU) admission, with an estimated risk of death up to 34% (1–4). This is likely due to the severe impairment of these patients’ immune systems, weakened by the hematological diseases themselves as well as their treatments, which results in ineffective cellular (5–7) and humoral (8–10) responses to SARS-CoV-2. This results in a lower rate of non-severe infection and immunization following COVID-19. Of note, although the deployment of vaccination has been of paramount importance for the general population, patients with hematological malignancies have also consistently shown lower rates of immunization following mRNA-based anti-SARS-Cov2 vaccination (11–13) and high rates of ICU admission and mortality following breakthrough infections (14). Although usually infection with omicron variants results in lower hospital admission rates and mortality, recent studies on outcomes in patients with hematological malignancies during the omicron wave still reported relatively high rates of mortality up to 9.1% and 16.5% in hospitalized patients (15, 16). These findings suggest that patients who fail to achieve a significant immunization following an infection, or a complete vaccination course need other preventive strategies, and could rely on passive immunization with neutralizing antibodies.

Tixagevimab/Cilgavimab (T/C), consists of a combination of two neutralizing anti-bodies against the SARS-CoV-2 surface spike protein which were developed from B-lymphocytes of patients affected by SARS-CoV-2. Thanks to the M252Y/S254T/T256E (YTE) and L234F/L235E/P331S (TM) modifications, T/C has a relatively long half-life of around 12 months and may thus be able to provide extended protection for up to one year after a single intramuscular administration. Its mechanism of action involves the simultaneous binding of tixagevimab and cilgavimab to two epitopes on the receptor binding domain of the virus spike protein (17). T/C was recently evaluated in the PROVENT trial in patients at increased risk of exposure to SARS-CoV-2 or inadequate response to vaccination (17). T/C led to an 82.8% decrease in the risk of developing symptomatic COVID-19 compared to placebo, with a favorable safety profile. These drugs were authorized by the European Medicines Agency (EMA) and by the Italian Drug Agency (AIFA) in March 2022 for pre-exposure prophylaxis in immunocompromised patients. However, the PROVENT trial included a limited number of patients with hematological malignancies and was conducted before the SARS-CoV-2 omicron variant became dominant (18). We thus designed a real-life study to assess whether T/C would be a viable passive immunization strategy in patients with hematological malignancies, that remained seronegative after SARS-CoV-2 vaccination.

## 2 Materials and methods

### 2.1 Study design

**Figure 1:**
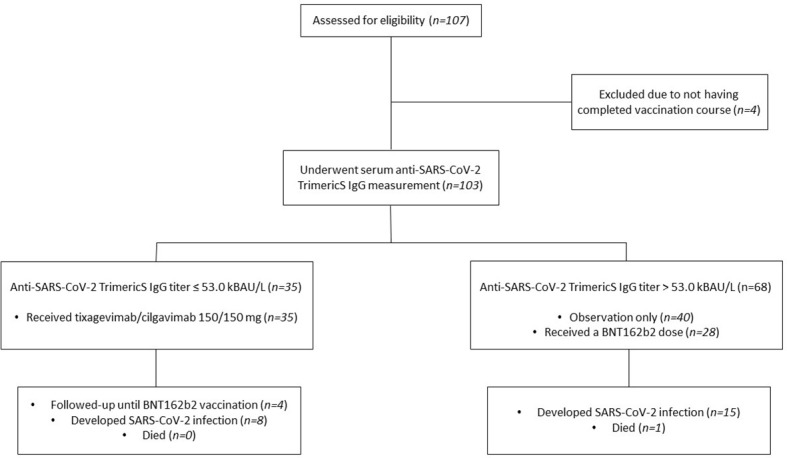
Study flow diagram.

In this prospective cohort observational study, we evaluated the efficacy of pre-exposure prophylaxis against SARS-CoV-2 with T/C in patients with hematological malignancies treated at the Hematology and Clinical Immunology Unit of Padova University hospital (**Figure 1**).

After a regular course of 3 doses of mRNA SARS-CoV2 vaccines, patients were as-signed, based on their serum anti-SARS-CoV-2 trimeric spike protein IgG titer, to receive either a 150/150 mg intramuscular injection of T/C or an observation only/vaccine arm. Patients with an IgG titer ≤ 53.0 kBAU/L received a single 150/150 mg dose of T/C, while patients with an IgG titer > 53.0 kBAU/L received either no intervention (observation only) or a fourth dose of BNT162b2 vaccine according to AIFA authorization. To be eligible for study inclusion, patients had to have received a previous full vaccination course with 3 doses of an mRNA SARS-CoV-2 vaccine. To limit potential bias, patients with a negative IgG titer that received a fourth BNT162b2 vaccine dose after the administration of T/C were censored at the time of vaccine administration and declared off-study. All seronegative patients had a negative nasopharyngeal swab within 7 days before T/C. Patients’ baseline clinical characteristics were obtained from electronic medical records at the time of anti-SARS-CoV-2 IgG titer measurement.

Patients were observed for the whole study period from March 17, 2022, until November 15, 2022. All subjects were followed from the date of T/C administration or an-ti-SARS-CoV-2 trimericS IgG titer measurement in the T/C and observation/vaccine groups respectively, until the development of SARS-CoV-2 infection or death. Patients not presenting the outcome of interest were censored at the time of last follow-up or at data cut-off (November 15, 2022). Patients were also monitored for the development of serious adverse events after T/C administration. The primary endpoint of the study was the cumulative incidence of SARS-CoV-2 infection confirmed by RT-PCR on a nasopharyngeal swab. In particular, we chose to report the 3-month cumulative incidence of survival as it was the most coherent time point with the median follow-up time for the whole cohort, and for both groups.

The study was conducted in accordance with The Strengthening the Reporting of Observational Studies in Epidemiology (STROBE) Statement and guidelines for observational cohort studies (19). The relevant STROBE checklist is included in the **Supplementary Material**.

### 2.2 Statistical analysis

According to data coming from the literature, the seroconversion rate after SARS-CoV2 vaccines is almost 67%, with a confidence interval of 95% and a margin error of 10%, the number of patients to test for anti-Spike antibody would have been at least 83. Considering a drop rate of 10%, the number of patients to recruit would have been at least 92. Baseline participants’ characteristics were compared with the χ2 test for categorical variables and with the student-t test or Kruskal-Wallis test for continuous variables, as appropriate. Median follow-up time for the whole cohort and for both treatment groups separately was calculated by the reverse Kaplan-Meier method. The cumulative incidence of infection in the two cohorts was estimated with the cumulative incidence function, treating death as a competing factor, and was compared with Gray’s test. Hazard ratios were computed by Fine-Gray regression. All p-values are two-sided and with a significance level of 0.05. R version 4.1.3 was used to conduct all statistical analyses and to plot cumulative incidence curves.

### 2.3 Anti-SARS-CoV-2 trimeric spike protein IgG measurement

Anti-SARS-CoV-2 trimericS IgG titers were measured in serum samples with DiaSorin LIAISON^®^ TrimericS IgG assay according to manufacturer instructions. LIAI-SON^®^ SARS-CoV-2 TrimericS IgG is an indirect chemiluminescent (CLIA) assay with high sensitivity and specificity for the detection of neutralizing IgG antibodies against the trimeric form of the SARS-CoV-2 spike protein (20–23).

## 3 Results

One hundred and seven patients were screened for eligibility, four were excluded due to not having completed a full vaccination course. One hundred and three patients were recruited in this study and underwent serum anti-SARS-CoV-2 trimericS IgG testing between March 17, 2022, and September 9, 2022. Baseline patients’ characteristics are summarized in **Table 1**.

**Table 1:** Baseline patients’ characteristics.

	Tixagevimab/Cilgavimab (n=35)	Observation only/Vaccine (n=68)	P
**Sex (%)**			0.51
M	18 (51)	41 (60)	
F	17 (49)	27 (40)	
**Age, mean (range)**	74.1 years (51 - 90)	63.5 years (23 - 85)	**< 0.001**
**Any hematological malignancy**	35 (100)	68 (100)	1
**Hematological malignancy (%)**			**0.005**
CLL	23 (66)	22 (32)	
NHL	9 (26)	16 (24)	
AML	2 (5.7)	4 (5.8)	
ALL	0	1 (1.5)	
MM	1 (2.9)	15 (22)	
AL Amyloidosis	0	3 (4.4)	
HCL	0	3 (4.4)	
HL	0	2 (2.9)	
MDS	0	1 (1.5)	
T-LGL	0	1 (1.5)	
**Number of treatment lines, median (range)**	2 (0 - 7)	1 (0 - 6)	0.15
**Actively receiving therapy (%)**			**0.01**
Yes	25 (71)	30 (44)	
No	10 (29)	38 (56)	
**Months on continuous therapy, mean (range)**	26.5 (1.4 - 69.3)	24.5 (0.69 - 94.1)	0.92
**Hypogammaglobulinemia (%)**			0.08
Yes	21 (60)	27 (40)	
No	14 (40)	41 (60)	
**Response to the last line of therapy (%)**			0.09
CR/VGPR	28 (80)	38 (56)	
PR	3 (11)	10 (14.7)	
SD	1 (2.9)	4 (5.9)	
PD	2 (5.7)	3 (4.4)	
Not evaluable	1 (2.9)	13 (19.1)	
**Previous SARS-CoV-2 infection (%)**			**0.004**
Yes	3 (8.6)	25 (36.8)	
No	32 (91.4)	43 (66.2)	
**Anti-SARS-CoV-2 trimericS IgG titer (%)**			**< 0.001**
≤ 53 kBAU/L	35 (100)	0	
> 53 kBAU/L	0	68 (100)	

Bold values, statistically significant p-values.

Thirty-five patients had an anti-SARS-CoV-2 trimericS IgG titer ≤53.0 kBAU/L and received 150/150 mg of T/C with a median time from antibody measurement to drug administration of 34 days (range 7-128). Sixty-eight patients had an anti-SARS-CoV-2 trimericS IgG titer > 53.0 kBAU/L and were assigned to the observation-only/vaccine group. Of those, 28 (41%) received a dose of the BNT162b2 vaccine during follow-up, with a median time from study inclusion to vaccination of 73.5 days (range 12-179). Only 4 patients (11%) in the T/C group received a BNT162b2 dose. Patients in the T/C group were older (mean age 74.1 vs 63.5 years, p<0.001), less frequently had previous COVID-19 (8.6% vs 36.8%, p < 0.001), were more commonly affected by chronic lymphocytic leukemia (66% vs 32%, p = 0.004) and less by multiple myeloma (2.9% vs 22%, p = 0.02) and a higher proportion of them was receiving active therapy (71% vs 44%, p = 0.01). The two cohorts did not statistically differ in terms of previous lines of therapy received for their disease, months on continuous therapy, response to the last therapy, or the rate of hypogammaglobulinemia.

**Figure 2:**
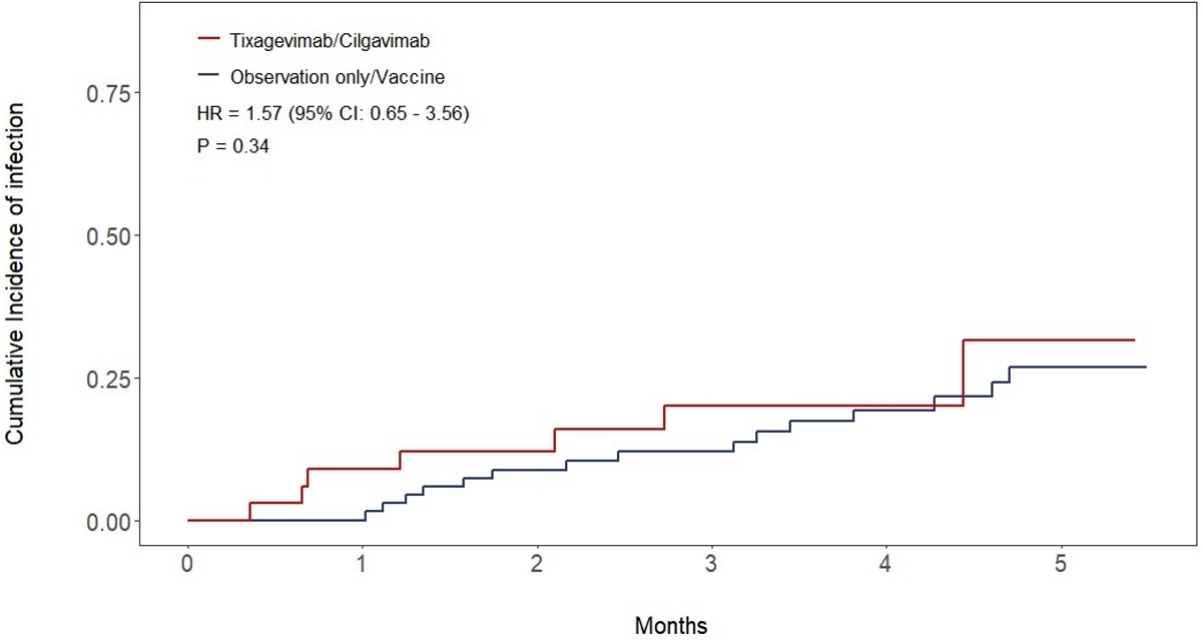
Cumulative incidence of infection curves for both cohorts.

All patients recruited in the study were included in the final analysis. Median follow-up was 4.24 months (IQR: 3.25-5.72) for the whole cohort, 3.88 months (IQR: 2.07-5.19) in the T/C group, and 5.06 months (IQR: 3.91-5.85) in the observation only/vaccine group. At data cutoff, a total of 8 patients (23%) in the T/C group contracted SARS-CoV-2 infection compared to 15 (22%) in the observation-only/vaccine group, no re-infections were observed. The estimated cumulative incidence of SARS-CoV-2 infection at 3 months was 20% (95% CI: 7.8-36%) in the T/C group versus 12% (95% CI: 5.6-21%) in the observation-only/vaccine group (HR 1.57; 95% CI: 0.65-3. 56; p = 0.34) (**Figure 2**). Regarding infection severity, all infections in both cohorts were either asymptomatic or mild except for one patient in the T/C group who required hospitalization and was treated with low-flow oxygen delivery and supportive care, without requiring intensive care unit admission. No fatalities due to SARS-CoV-2 were observed. A single death was recorded during follow-up and occurred in the observation only/vaccine group in a patient who did not contract the infection.

Among patients that were currently receiving therapy for their hematological malignancy, the 3-month cumulative incidence of SARS-CoV-2 infection was 24% (95% CI 8.1 – 44%) in the T/C group versus 6.8% (95% CI 1.2 – 20%) in the observation only/vaccine group (HR 1.52; 95% CI: 0.53-4.32; p = 0.44). Among those not currently receiving therapy, the 3-months cumulative incidence of infection was 11% (95% CI 0.47 – 41%) in the T/C group versus 16% (95% CI 6.4 – 30%) in the observation only/vaccine group (HR 1.43; 95% CI: 0.28-7.15; p = 0.67). When excluding patients that received a fourth vaccine dose from the analysis, the 3-months cumulative incidence of SARS-CoV-2 infection was 22% (95% CI: 8.4-39%) versus 18% (95% CI: 7.8-32%) in the T/C and observation only groups respectively (HR 1.13; 95% CI: 0.46-2.77; p = 0.79). No statistically significant difference in the incidence of SARS-CoV-2 infection was also found when analyzing the subgroup of patients affected by chronic lymphocytic leukemia, which was the most common hematological malignancy in our study (21% vs. 9.1% in the T/C and observation only/vaccine groups respectively; HR 1.68, 95% CI 0.51-5.56, p = 0.4).

## 4 Discussion

Our data show that pre-exposure prophylaxis with T/C resulted in similar incidences of SARS-CoV-2 infection in seronegative patients compared to seropositive patients who followed a different prevention strategy. Since our study was conducted during the omicron surge, this is not unexpected as there is evidence that T/C has limited neutralizing capacity against omicron subvariants (24, 25). However, as the treated group consisted of seronegative patients who did not respond to vaccination and with a supposed higher risk of SARS-CoV-2 infection, our real-world data suggest that T/C may still have provided some degree of protection, comparable at least to that given by previous seroconversion and a higher anti-SARS-CoV-2 IgG titer. This is however difficult to demonstrate given that no data exists demonstrating a higher incidence of SARS-CoV-2 infection in seronegative patients with hematological malignancies, compared to seropositive ones. Thus, this result has to be interpreted in light of this limitation of our study design, in which two different groups with likely different baseline risks were compared. Also, the observational nature of the study and the lack of randomization must be considered. When the study was conducted, by AIFA regulations T/C could be administered only to patients with an anti-SARS-CoV-2 trimeric spike protein IgG titer ≤ 53.0 kBAU/L. Thus, seropositive patients could not receive T/C, and not administering T/C to seronegative patients would have been unethical. This hindered the creation of two perfectly homogeneous cohorts. Other limitations that must be considered in our study include the fact that our study was a pilot study with a low number of subjects, and the relatively short follow-up time, which makes it impossible to assess any long-term effect of T/C.

The subgroup analyses showed a trend towards a higher risk of infection in the T/C group when restricting the analysis to those patients who were currently receiving therapy directed at their hematological malignancy, albeit not statistically significant. This result possibly points to the fact that T/C may have been less effective in patients receiving active therapy. As expected, patients not receiving therapy had lower incidences of infection. In-deed, the differences in baseline characteristics between the two cohorts may also have played a role in the outcome of the study. In particular, the higher age coupled with a higher proportion of patients receiving active therapy in the T/C cohort posed this population at a higher risk for infection and severe COVID-19. As stated, this higher risk may have been only partially mitigated by T/C. Indeed, a large study recently identified higher age as independently associated with a worse outcome after SARS-CoV-2 infection in patients with hematological malignancies (26). In particular, age likely played a role in the infection rate and development of immunity, as demonstrated by the fact that all patients in the T/C group failed seroconversion. As the aging immune system is more susceptible to infections due to immunosenescence, it is thus possible that the T/C group that consisted of slightly older individuals could be even more at risk of infection. As discussed above, T/C, may might have partially mitigated this risk. The fact a single hospitalization was observed in the T/C group compared to none in the observation-only/vaccine one, may also arise from the T/C cohort bearing a higher baseline risk. While this could also reflect a lower ability of T/C to protect from more severe forms of COVID-19, this difference is too minimal to draw meaningful conclusions. The possibility of receiving a fourth vaccination in the observation-only/vaccine group apparently did not impact significantly on the incidence of SARS-CoV-2 infection and excluding these patients from the alter the result of the primary outcome significantly, especially in a short time frame of 3 months.

Since Feb 2022, FDA recommended increasing the dose from 150/150 mg to 300/300 mg based on data suggesting the higher dose would be more likely to prevent infection by omicron variants, and also due to the low availability at that time of other monoclonal antibodies such as sotrovimab. However, this dosage is still not approved by AIFA and none of our patients received the higher dose. A higher dose of 300/300 mg may have been able to provide more protection, thus lowering the incidence of infection in patients treated with T/C. Other studies that have explored the efficacy of T/C in patients with hematological malignancies utilized both dosages. In studies using the 300/300 mg dose, the rates of SARS-CoV-2 infections after T/C ranged from 11% to 9.3% (27, 28). In studies using the 150/150 mg dose, the same rate ranged from 28% to 14% (29, 30). Notwithstanding the important limitations of cross-study comparisons, these percentages hint at a trend to-wards higher incidences of infections in patients treated with the lower dose, and one may speculate on whether the 300/300 mg dose would have resulted in lower infections in the T/C group. However, since this variation appears limited, we believe that a higher dose would not have resulted in significantly reduced rates of infections in the T/C cohort compared with the observation-only/vaccine one. To this date, no studies that compared the two dosages exist, and this remains an open question.

Another prospective study on the use of T/C in patients with hematological malignancies produced results comparable to our experience. In a larger cohort of 203 patients treated with T/C, the incidence of symptomatic SARS-COV-2 infection was 9.3%, with only one patient requiring hospitalization, and a local incidence of infection comparable to that of the general population (11.3%) (27). Compared to this work by Ocon and colleagues, the incidence of SARS-COV-2 infections was higher in our cohort as we included also asymptomatic infections. Similar evidence for the use of pre-exposure prophylaxis with T/C comes from another recent multicentre retrospective study, which evaluated T/C in 161 recipients of allogeneic hematopoietic stem cell transplant during the omicron wave and observed a similar (14%) rate of COVID-19, without cases of severe infections (29). Overall, these data are in keeping with our results and reinforce the hypothesis that T/C could provide added protection to patients with hematological malignancies. Despite not being complete, this protection may reduce the incidence of infection to a level comparable to that of the immunized population of patients with hematological malignancies. Interestingly, a recent report on a small number of patients has also highlighted the potential therapeutic role of T/C in patients with hematological malignancies who failed to respond to vaccination, with early administration during asymptomatic infection from SARS-COV-2 Omicron variant possibly halting the development of severe COVID-19 (31).

Our results also underline that despite treatment with T/C or a seropositive state, patients with hematological malignancies are still at risk of breakthrough SARS-CoV-2 infection, as highlighted also by a recent retrospective study that evaluated T/C in patients with B-cell malignancies (28) and by another study that reported a 3.8% rate of symptomatic COVID-19 in a cohort of 54 patients treated with T/C after a median follow up time of 74 days (18). Applying continuous vigilance along with all means of prevention is thus still a priority in patients with hematological malignancies.

Regarding safety, serious adverse events appear to be a rare occurrence with T/C. In studies reporting on the use of T/C for prophylaxis in the general population, the rate of serious adverse events appears low, with a reported rate of serious adverse events of 1.8% in the original phase 3 trial (15). Other studies involving patients with hematological malignancies that recorded adverse events with T/C, reported mostly low-grade adverse events including diarrhoea and rash, with only one serious adverse event reported thus far (27–29). Overall, including our reported cohort, this accounts for a single serious adverse event observed in a total of 636 patients either with hematological malignancies or subjected to allogeneic stem cell transplantation, treated with T/C.

The use of T/C for pre-exposure prophylaxis has also been investigated in other populations not affected by hematological malignancies, yet still immunocompromised. A retrospective study, also conducted during the omicron wave, in 444 solid organ trans-plant recipients found a statistically significant lower rate of infection in patients who received T/C versus those who did not (5% vs 14%, p < 0.001). Interestingly, in this work, the authors found that the 150/150 mg dose was associated with a higher incidence of break-through infections (p = 0.025) (32). Another multicentre cohort study evaluated the efficacy of T/C in a large cohort of 1112 immunocompromised patients. With a relatively short median follow-up of 63 days, the incidence of COVID-19 was 4.4%. The mean weekly incidence rate of infection was also lower than that of the general population in patients that received T/C (33). Other similar works involving immunocompromised individuals, and kidney and heart transplant recipients reached similar conclusions with observed incidences of SARS-CoV-2 infection ranging from 3.5 to 14.7%, and overall better outcomes after infection (34–37). These results from other immunocompromised populations appear in line with what has been observed in patients with hematological malignancies, pointing at least to a partial benefit of T/C administration in high-risk individuals. Evidence about the effectiveness of T/C comes also from two systematic reviews and meta-analyses, that confirmed the efficacy of pre-exposure prophylaxis with T/C (38, 39). The results from these meta-analyses may however be not fully generalizable to the omicron wave, as they aggregate studies from different periods of the pandemic.

A limitation to the future use of T/C is the newer emerging SARS-CoV-2 variants. As with many other viruses, SARS-CoV-2 progressively acquires new sets of mutations that alter the virus characteristics and lead to the emergence of different variants and subvariants. When these mutations occur in the spike protein gene, the neutralizing ability of monoclonal antibodies directed to it, such as T/C, is severely reduced. As stated before, the reduced activity of T/C against Omicron subvariants BA.1 and BA.2 has already been re-ported (24, 25), and as time passes and new subvariants emerge, the efficacy of T/C is at stake. Indeed, as of 26 January 2023, the FDA retired the emergency use authorization for T/C, since it may not retain its activity against newly emergent omicron subvariants, and to prevent unnecessary exposure to possible serious side effects such as allergic reactions. As of the same date, the use of T/C is still approved in Europe, although the EMA’s emergency task force has cautioned that T/C and other monoclonal antibodies are unlikely to be effective against emerging strains.

In light of SARS-CoV-2’s ability to evade monoclonal antibodies directed to the spike protein, new antibody designs with different mechanisms of action may overcome this issue. Of such antibodies, SP1-77 acts by inhibiting viral-host membrane fusion and demonstrated significant SARS-CoV-2 neutralization potential through variants BA.5 (40). Other strategies to develop monoclonal antibodies could minimize the likelihood of resistance by targeting either conserved epitopes on the SARS-CoV-2 receptor binding do-main or susceptible ones on new variants through the study of mutational patterns (41, 42). Moreover, new antibodies destined for immunocompromised patients are in clinical development and built on the efficacy profile shown by T/C before the Omicron wave. As a significant example, AZD5156, which combines the T/C component cilgavimab with the new investigational long-acting monoclonal antibody AZD3152 entered clinical testing with the phase I/III trial SUPERNOVA (NCT05648110).

Taken together, the results of our study coupled with the available evidence from the literature may suggest a moderate benefit of pre-exposure prophylaxis with T/C. However, this benefit and its eventual extent are difficult to demonstrate, as randomized clinical trials comparing T/C versus no intervention are currently hardly feasible. In light of the hypothesized lack of activity of T/C against newer omicron variants, it is likely that its use will decline unless new real-life evidence emerges. Studies comparing T/C with other interventions in homogeneous patient populations and conducted in more recent times may provide further insights. Taking a look at beginning of the omicron wave during which our study was conducted, however, even a small contribution of T/C in lowering the rates of infection, hospitalizations, and ICU admissions for a fragile population of patients with hematological malignancies could have proved significant for these individuals. Given the good safety record, the risk/balance benefit was probably in favor of T/C while it could be used against sensitive variants. Whether this remains true at present times, and whether T/C will continue to be employed in immunocompromised patients, is yet to be seen and depends on the physicians’ and regulatory agencies’ decisions. Although T/C may not remain a viable option for SARS-CoV-2 prevention in immunocompromised patients with hematological malignancies, the results from our work may still help in the design of other monoclonal antibodies and in structuring future prevention strategies. SARS-CoV-2 is unlikely to disappear, and new infective agents are likely to come in the future. In addition, a novel long-acting anti-SARS-CoV-2 monoclonal antibody, AZD5156, has been developed and is able to neutralize BQ.1 and BC1.1 variants *in-vitro*. The phase I/III clinical trials SUPERNOVA (NCT05648110) investigating pre-exposure prophylaxis with AZD5156 in immunocompromised patients and/or less likely to mount an adequate protective response after the SARS-CoV-2 vaccine is ongoing. Our and other studies on monoclonal antibodies-based pre-exposure prophylaxis might hopefully help in the organization and management of future SARS-CoV2 waves and other pandemics.

## Data availability statement

The raw data supporting the conclusions of this article will be made available by the authors, without undue reservation.

## Ethics statement

The studies involving human participants were reviewed and approved by Padova University Hospital local research ethics committee (number 4430/AO/18). The patients/participants provided their written informed consent to participate in this study.

## Author contributions

Conceptualization, AV and LT. Methodology, data curation, formal analysis, investigation, methodology, writing, FA and MP. Resources TB, GBi, GBo, AB, MC, CC, AC, FD’A, LF, IG, CG, SI, FL, AM, FM, LP, SPe, FP, SPr, VR, GS, FV, RZ, IZ, SZ. Validation, AV. All authors contributed to the article and approved the submitted version.
